# Preliminary screening of new biomarkers for sepsis using bioinformatics and experimental validation

**DOI:** 10.1371/journal.pone.0317608

**Published:** 2025-01-24

**Authors:** Hao Wang, Wei Xiong, Wu Zhong, Yingchun Hu

**Affiliations:** 1 Clinical Medical College, Southwest Medical University, Luzhou, People’s Republic of China; 2 Department of Emergency Medicine, The Affiliated Hospital of Southwest Medical University, Luzhou, People’s Republic of China; University of Illinois Urbana-Champaign, UNITED STATES OF AMERICA

## Abstract

**Background:**

The morbidity and mortality of sepsis remain high, and so far specific diagnostic and therapeutic means are lacking.

**Objective:**

To screen novel biomarkers for sepsis.

**Methods:**

Raw sepsis data were downloaded from the Chinese National Genebank (CNGBdb) and screened for differentially expressed RNAs. Key genes with predictive value were identified through weighted correlation network analysis (WGCNA) and meta-analysis and survival analysis using multiple public databases. Core genes were analyzed for functional enrichment using Gene Set Enrichment Analysis(GSEA). The core genes were localized using single-cell sequencing. qPCR was used to validate the core genes.

**Results:**

Differential analysis yielded a total of 5125 mRNA. WGCNA identified 5 modules and screened 3 core genes (S100A11, QPCT, and IFITM2). The prognosis of sepsis patients was strongly linked to S100A11, QPCT, and IFITM2 based on meta-analysis and survival analysis(P < 0.05).GSEA analysis showed that S100A11, QPCT, and IFITM2 were significantly enriched in ribosome-related pathways. S100A11 and QPCT were widely distributed in all immune cells, and QPCT was mainly localized in the macrophage cell lineage. In the sepsis group, the qPCR results showed that S100A11, QPCT, and IFITM2 expression levels were significantly higher in the sepsis group(P < 0.05).

**Conclusion:**

In this study, S100A11, QPCT, and IFITM2 were screened as new potential biomarkers for sepsis. Validated by bioinformatics analysis and qPCR, these genes are closely associated with the prognosis of sepsis patients and have potential as diagnostic and therapeutic targets.

## Introduction

Sepsis is a systemic response to infection with complex pathophysiologic mechanisms involving interactions at multiple cellular and molecular levels [1]. Sepsis, a frequent complication in critically ill individuals, is the primary reason for mortality in patients globally, resulting in approximately 50 million cases and 11 million fatalities each year [2]. Sepsis is a major contributor to high death rates worldwide, particularly in ICU settings [3]. As the symptoms of sepsis are similar to those of several diseases, this makes its early diagnosis complex and difficult, and delays in treatment significantly increase the risk of death in patients [4]. Therefore, the search for new biomarkers for early diagnosis and disease surveillance is an important direction of current research.

In cellular physiology, ribosomes are the site of protein biosynthesis and play a key role in maintaining normal cellular function and responding to environmental stresses [5–7]. Under conditions of infection and inflammation, the function of ribosomes may be affected, thereby altering the pattern of protein synthesis, which may affect cell survival and function [8]. These scientific findings suggest that ribosomes play an important role in the inflammatory response and immune regulation in sepsis and may provide new insights into the diagnosis and treatment of sepsis. About 60% of approved antibiotics discovered to date fight pathogenic bacteria by targeting the ribosome [9,10]. Furthermore, ribosomal protein L13 is involved in the innate immune response triggered by the foot-and-mouth disease virus, highlighting the importance of ribosomal protein L13 in viral infection and immune system control, which predicts that it may play a key role in the diagnosis and treatment of sepsis [11].

**Fig 1:**
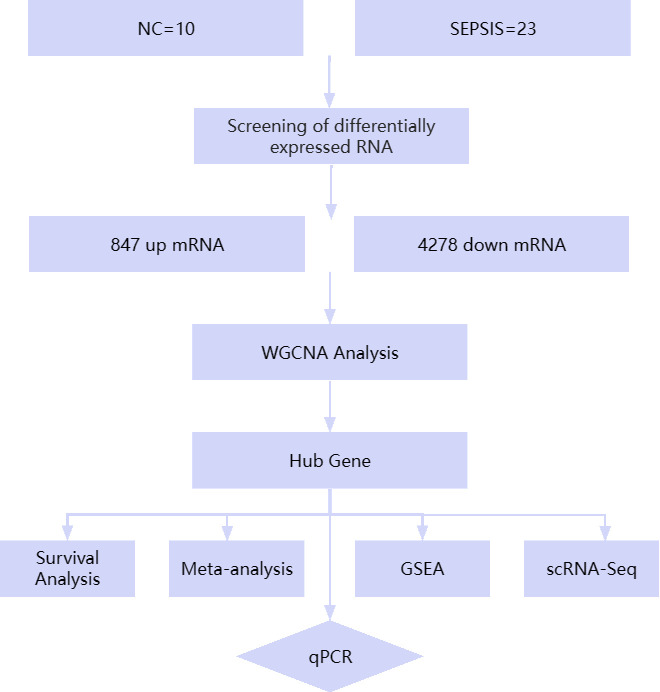
Flowchart of this study. Exploration of new biomarkers for sepsis using bioinformatics and qPCR experimental validation methods. NC, normal control; WGCNA, Weighted Gene Co-Expression Network Analysis; GSEA, Gene Set Enrichment Analysis; scRNA-Seq, Single-Cell RNA Sequencing.

Based on the above analysis, given the key role of ribosomes in inflammation and immune response, we chose three genes, S100A11, IFITM2, and QPCT, which are closely related to ribosomal function, as the focus of analysis in this study to explore their role and potential diagnostic value in sepsis. By analyzing in detail the expression patterns of these genes in sepsis patients and their associations with the prognosis of sepsis patients, this study aims to reveal the possibility of these genes as de novo markers of sepsis, laying the foundation for further research on the regulatory mechanisms of S100A11, IFITM2, and QPCT in sepsis and their clinical significance, and providing the possibility of developing new therapeutic targets for sepsis. Fig 1 displays the study’s flow diagram.

## Methods

### Sample collection

The research team at the Affiliated Hospital of Southwest Medical University conducted the collection from February 2019 to December 2020, following approval from the hospital’s ethics committee and adhering to the principles of the Declaration of Helsinki [12], and obtained verbal consent from 23 septic patients and 10 healthy volunteers who participated in this study. Every individual with sepsis was recognized based on the most recent sepsis 3.0 diagnostic standards, and blood specimens were obtained within the first 24 hours of being admitted. Samples from healthy volunteers were obtained during routine physical examinations during the same period. Exclusion criteria: (1) age less than 16 years or more than 80 years; (2) previous organ failure or immune system disease or hematologic disease; (3) the patient or the patient’s family or the patient’s family member has suffered from organ failure or immune system disease or hematologic disease; (4) the patient or family member refused to participate in the study. Blood samples were collected using PAXgene tubes Blood samples were collected according to the company’s manual and human blood samples were stored in the hospital’s biospecimen bank. (Ethical approval number: ky2018029, clinical trial number: ChiCTR1900021261, Registration date: February 4, 2019.).The raw data was submitted to CNGBdb under project code CNP0002611.

### Analysis of RNA with varying levels of expression

Before analyzing the transcriptome data with the iDEP96 web tool [13], a rigorous quality control procedure was carried out to verify the data accuracy. Principal component analysis (PCA) was then employed to reduce dimensionality and detect potential outlier samples to ensure the accuracy and reliability of the data analysis. DEseq2 was utilized to conduct differential expression analysis, with a minimum fold change (FC) threshold of 2 and a false discovery rate (FDR) requirement of less than 0.05 to identify RNAs showing significant differential expression.

### WGCNA and co-expression networks

WGCNA, also known as Weighted Gene Co-Expression Network Analysis, is a computational approach used to identify relationships between genes in microarray data [14].In this study, we first constructed a matrix of gene expression data and calculated the correlations between genes as a basis for generating a weighted neighbor-joining matrix, followed by adjusting the soft threshold β to strengthen the connections between highly correlated gene pairs. In addition, a topological overlap matrix (TOM) was constructed using the weighted neighbor-joining matrix, and then the indirect interactions between genes were analyzed. Finally, applying a hierarchical clustering approach, we identified several tightly connected gene modules. The key genes were screened by constructing co-expression networks for the top 30 genes within the key modules using Cytoscape 3.10.1 [15].

### Survival curve analysis

In this study, we analyzed the association between gene expression and prognosis in sepsis patients using the public dataset GSE65682 [16]. The dataset includes transcriptomic data from whole blood of 479 patients with sepsis, as well as information on the survival of these patients after 28 days. By ordering the 293 assessed genes from low to high expression, we categorized these genes into a low-expression group and a high-expression group, with 50% each. Survival times in both groups were analyzed statistically, with a significance level of p < 0.05 used to determine statistical significance. Furthermore, this research analyzed the variations in gene expression of important genes in both sepsis and non-sepsis groups to investigate the potential significance of these genes in disease prognosis.

### Meta-analysis

In this study, a Meta-analysis was used to assess the differences in biomarkers between sepsis survivors and non-survivors [17].We obtained and evaluated sepsis datasets GSE54514 [18], GSE63042 [19], and GSE95233 [20] from the GEO database (https//www.ncbi.nlm.nih.gov/geo/). The choice of datasets was guided by research with a reasonable sample size, ensuring uniform experimental design and data integrity. The screened datasets were preprocessed and standardized by indicators such as sample size, mean, and standard deviation to ensure the comparability of data between different studies. Subsequently, a random-effects model was employed to combine the variations in core gene expression among studies, considering the potential diversity among them. Meta-analysis statistical significance tests were conducted using a modified Q-value and I2 statistic to evaluate the impact of heterogeneity.

### GSEA enrichment analysis

GSEA is a computational technique that evaluates if a specific group of genes shows notable variations across various biological conditions [21].In this study, the analysis was performed using R language version 4.3.2, which first defines predefined gene sets based on these genes and calculates the enrichment score for each gene set to reflect whether the gene sets show statistically significant differences in expression between different biological states. We conducted numerous random permutation tests to determine the null hypothesis distribution of the scores and calculate a normalized enrichment score (NES) for each gene set. The NES was adjusted for multiple tests using the FDR to detect significantly enriched gene sets. Furthermore, we delved deeper into the functions of these highly enriched gene sets in biological processes, cellular components, and molecular functions using gene ontology (GO) analysis and Kyoto Encyclopedia of Genes and Genomes (KEGG) pathway analysis [22–24].

### Single-cell sequencing

This research utilized 10x Genomics single-cell sequencing technology to examine five peripheral blood samples from individuals without health issues, individuals with Systemic Inflammatory Response Syndrome (SIRS), and individuals with sepsis. This study aimed to pinpoint the localization of specific target genes in different cell types to provide the necessary cellular localization information for future in vitro functional studies. We utilized Cell Ranger and Seurat software to conduct quality assessment and data manipulation on the high-throughput sequencing data, which involved performing PCA and t-distribution random neighbor embedding (tSNE) for visualizing the data. In addition, the FindAllMarkers and feature map functions in the Seurat package were utilized in this study to identify and visualize the cell-specific expression of marker genes, further deepening the understanding of target gene cell lineage localization.

### Cell culture and modeling of LPS inflammation

Human monocytic leukemia THP-1 cells were utilized as the experimental model in this research. The experiment involved utilizing RPMI-1640 base medium for cell cultivation, supplemented with 10% FBS and 1% P/S, and then placing it in a sterile environment at 37°C with 5% CO2 and saturated humidity. To induce THP-1 cell differentiation to macrophages, we first inoculated 3.0 ×  10^5^ normal THP-1 cells in 6-well plates and treated them with 50 ng/mL of PMA for 24 hours. Following this, the previous medium was substituted with 1.5 mL of new serum-free growth medium. Then, 100 pmol of siRNA directed against S100A11, IFITM2, and QPCT were introduced into serum-free, antibiotic-free Opti-MEM® Medium, combined with 4 microliters of Lipo8000™ Transfection Reagent, and left to sit for 20 minutes at ambient temperature. The mixture was added to the cells and the medium was changed after 5–6 hours. Following a 24–hour transfection period, the cells were stimulated with 100 ng/mL of LPS for 6 hours.

### Q-PCR experiments

Q-PCR is a highly sensitive technique for the simultaneous amplification and quantification of target DNA molecules, which enables precise measurement of the amount of specific DNA sequences in a sample by using fluorescence to monitor the amplification process and is commonly used to quantify the expression level of specific genes in a sample [25]. This study conducted in vitro experiments using the THP-1 human monocytic leukemia cell line to investigate the expression patterns of key genes in sepsis. RNA extraction was first performed using RNAiso Plus reagent, followed by centrifugation by adding chloroform and controlling the temperature at 4°C, and RNA precipitation with isopropanol. The precipitated RNA was washed with ethanol, dried, and resuspended in RNA-free water. Subsequently, genomic DNA contamination was removed using gDNA Eraser, followed by cDNA synthesis by PrimeScript™ RT Master Mix to provide templates for qPCR analysis. The qPCR was performed by setting appropriate reaction conditions, including pre-denaturation and cyclic amplification steps, and finally, the target gene expression was quantified by the 2^-⊿⊿^^method^. The experimental results were visualized using R language 4.3.2.

## Results

### Screening for differentially expressed RNA

**Fig 2:**
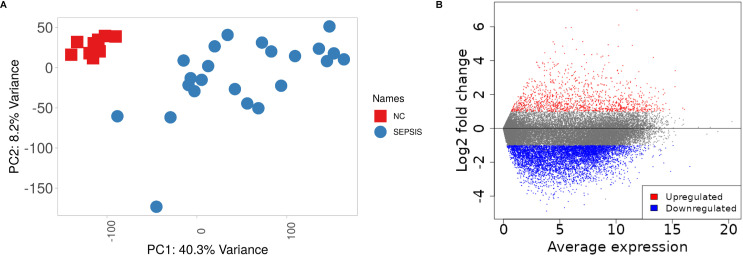
Displays the analysis of RNAs with varying expression levels. A: PCA plot demonstrates distinct clustering between the NC and SEPSIS categories, with no samples that deviate from the expected pattern. B: The volcano plot displays RNAs that are up-regulated in red and down-regulated in blue.

PCA analysis showed strong uniformity and clear distinction between normal control and sepsis samples, with no outliers detected (Fig 2A). After screening for | FC|≥2 and selecting FDR < 0.05, a total of 5125 mRNAs were found to be differentially expressed in this research, with 847 showing up-regulation and 4278 showing down-regulation. Fig 2B demonstrates the results of differential expression analysis.

### Identification of WGCNA and core genes

**Fig 3:**
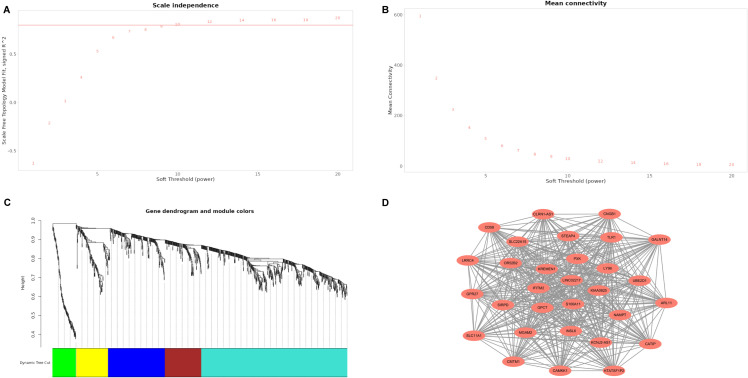
WGCNA as well as the co-expression network. A: Module topology fit gradually increases with increasing soft threshold. B: Average connectivity gradually decreases with increasing soft threshold. C: Modules based on overlapping co-expression topologies of different colored mRNAs (module size > 50). The blue module is significantly associated with the clinical features of sepsis. D: There are 30 genes in this network, with S100A11, QPCT, and IFITM2 located in the center of the network.

A threshold of 9 was chosen in the WGCNA study by observing the soft threshold trend

(Fig 3A–B). The analysis revealed five modules of size 50 each, grouped according to gene expression similarities. The blue module was found to have a strong correlation with sepsis phenotype (Fig 3C). Following this, Cytoscape version 3.10.1 was utilized to build a co-expression network of the leading 30 genes in their respective modules, with S100A11, QPCT, and IFITM2 positioned at the core of the network as shown in Fig 3D.

### Survival curve analysis

**Fig 4:**
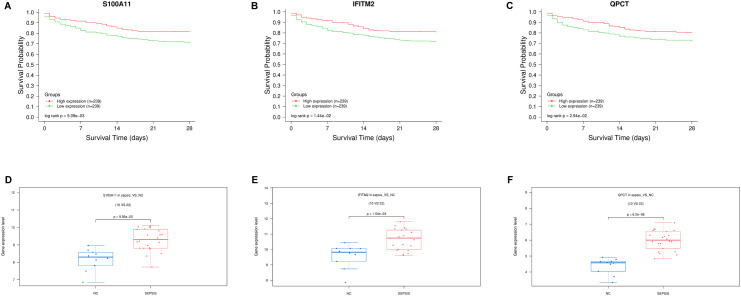
Displays the prognostic analysis and expression of important genes, with survival time in days on the horizontal axis and survival rate on the vertical axis. A–C: Low mRNA samples are indicated by the green line, while high mRNA samples are represented by the red line, both with p-values below 0.005. D–F: S100A11, QPCT, and IFITM2 were highly expressed in the sepsis group.

To further analyze the correlation between key genes and the prognosis of sepsis, the present study combined with the public database GSE65682 [16] to analyze the survival curve of the above key genes. The findings indicated a positive correlation between the survival of sepsis patients and the levels of S100A11, QPCT, and IFITM2 expression (Fig 4A–4C). These three genes are involved in the prognosis of sepsis patients and may be novel targets. Furthermore, the sepsis group exhibited high expression levels of S100A11, QPCT, and IFITM2 as shown in

Fig 4D–4F.

### Meta-analysis

**Fig 5:**
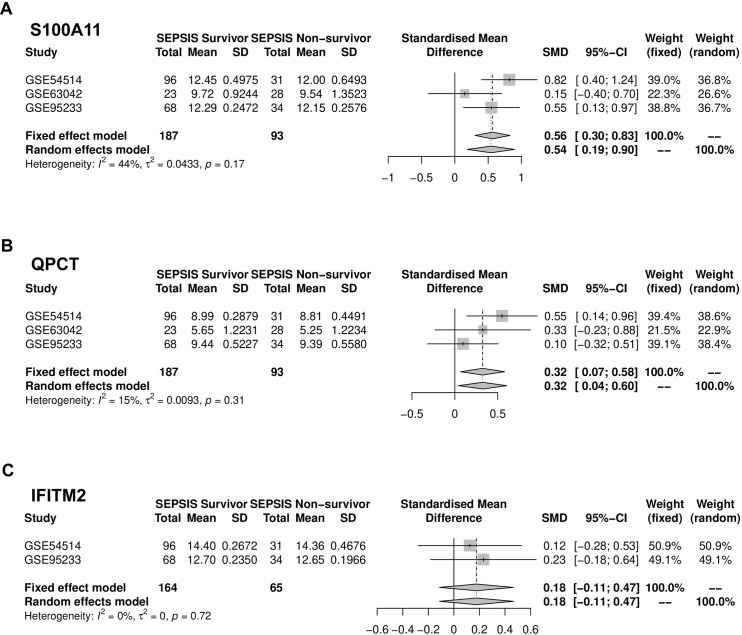
Meta-analysis results. A meta-analysis was conducted comparing the levels of S100A11, IFITM2, and QPTC expression between the sepsis survivors and non-survivors using the GSE54514, GSE95233, and GSE63042 datasets. The I^2^ value is 44% for the S100A11 and 15% and 0% for the QPCT and IFITM2, respectively.

The meta-analysis findings indicated variations in the levels of S100A11, QPTC, and IFITM2 expression among sepsis patients who survived and those who did not (Fig 5). In Meta-analysis, we assessed heterogeneity. For the analysis of the S100A11 gene, the I^2^ value was 44%, indicating moderate heterogeneity, while the QPCT and IFITM2 genes showed low heterogeneity with I^2^ values of 15% and 0%, respectively. The standardized mean difference (SMD) for S100A11 gene expression between survivors and non-survivors was 0.56 (95% CI [0.30, 0.83]), signifying a significant contrast. QPTC gene expression had an SMD of 0.32 (95% CI [0.07, 0.58]), suggesting a distinction between survivors and non-survivors. The SMD for the IFITM2 gene was 0.18 (95% CI [−0.11, 0.47]), indicating a smaller difference. These results suggest that the expression of S100A11, IFITM2, and QPTC is strongly associated with the survival of septic patients.

### GSEA enrichment analysis

**Fig 6:**
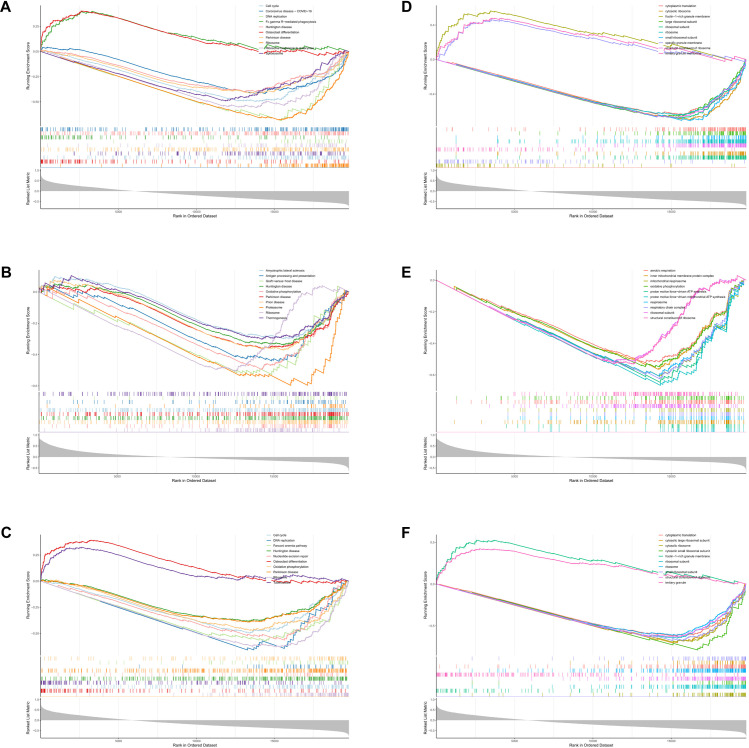
Results of GSEA enrichment analysis. A-F: The spikes in the enrichment score (ES) curves reveal a tendency for these gene sets to concentrate in the sequenced gene list, suggesting their potential activity in the study state. The results of the GSEA enrichment analysis indicate that S100A11, QPCT, and IFITM2 are closely related to ribosome function.

In this study, KEGG pathway analysis of S100A11, QPCT, and IFITM2 showed that the ribosome-related pathway exhibited significant enrichment. These results suggest that these three genes may play key roles in ribosome function, especially during protein biosynthesis (Fig 6A–6C). In addition, significant enrichment of S100A11, QPCT, and IFITM2 in biological processes and molecular functions related to ribosome function was also observed by GO analysis, which further emphasized their importance in regulating ribosome activities and their role in cellular physiological and pathological processes (Fig 6D–6F). These findings reveal that S100A11, QPCT, and IFITM2 may play important roles in the pathophysiological processes of sepsis.

### Single-cell sequencing

**Fig 7:**
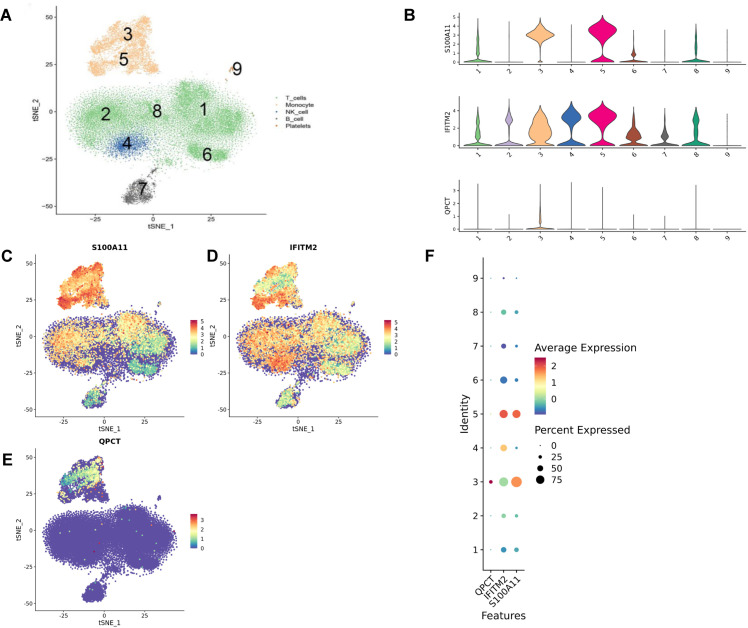
The localization of important genes in cell lineage is depicted. A: Macrophages are found in Groups 3 and 5, natural killer cells in Group 4, T cells in Groups 1, 2, 6, and 8, B cells in Group 7, and platelets in Group 9. B-E: The distribution of S100A11 and IFITM2 is widespread among all immune cells, while QPCT is mainly found in macrophages. F: Each bubble in the figure represents the average gene expression in a specific cell population, with the size of the bubble indicating the percentage of cells expressing the gene in that population.

After analyzing the transcriptome sequencing of the mentioned five samples, nine cell groups were identified through clustering after reducing dimensions. Macrophages are represented by 3 and 5, natural killer cells by 4, T cells by 1, 2, 6, and 8, B cells by 7, and platelets by 9 (Fig 7A). Single-cell localization of S100A11, QPCT, and IFITM2 revealed that S100A11 and IFITM2 were widely distributed in all immune cells, and QPCT was mainly localized in the macrophage lineage (Fig 7B–F).

### Q-PCR experiments

**Fig 8:**
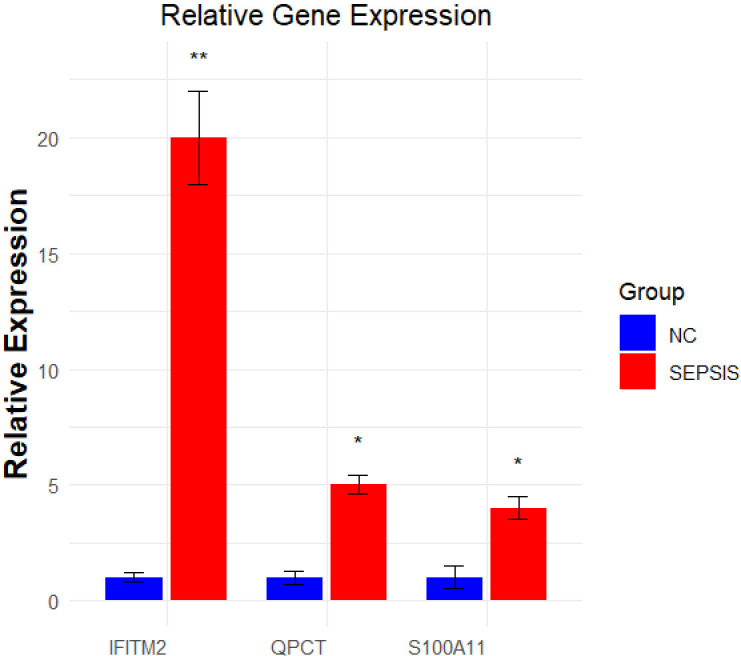
Results of qPCR experiments. qPCR detects the expression of three core genes in the sepsis cell model. The blue color indicates the control group and the red color indicates sepsis. *p < 0.05; **p < 0.01.

In a q-PCR assay, this study compared the gene expression differences between the sepsis group and the healthy control group. The qPCR test revealed a notable increase in the expression of S100A11, QPCT, and IFITM2 in the sepsis group compared to the control group, as depicted in Fig 8. This indicates that the control of S100A11, QPCT, and IFITM2 expression could be significant in the development of sepsis. Table 1 displays the specific primer sequences.

**Table 1:** Primer sequences.

Primer Name	Sequence (5’–3’)
S100A11-F	TTGACCGCATGATGAAGAAACT
S100A11-R	ATGACAGAAAGGCTGGAAGGAA
IFITM2-F	GTCCCTGTTCAACACCCTCTTC
IFITM2-R	CCAACCATCTTCCTGTCCCTAG
QPCT-F	CATTGCTGATAGAGCGATACCC
QPCT-R	TCATAGTGGCAGGCGAGGAC

## Discussion

The present study revealed the expression pattern, cell line localization, and functional enrichment of S100A11, IFITM2, and QPCT in macrophages, aiming at an in-depth exploration of the roles of S100A11, IFITM2, and QPCT in sepsis to evaluate their potential as novel biomarkers.

S100A11 is a member of the S100 family that mediates signaling in response to internal or external stimuli and plays a variety of roles in diseases as diverse as cancer, metabolic diseases, neurological disorders, and vascular calcification [26]. S100A11 can be involved in controlling calcium signaling and cell growth and has been identified as playing a part in various inflammatory and immune reactions [26]. Meanwhile, existing studies also suggest that S100A11 has the potential to be a potential carcinogen and prognostic marker associated with the pan-cancer immunosuppressive microenvironment [27]. Additionally, IFITM proteins are now known to be involved in regulating immune responses, such as innate antiviral and inflammatory responses, as well as adaptive t-cell and b-cell responses [28]. Among them, IFITM2 has been extensively investigated for its role in antiviral and anti-microbial infections [29]. Specifically, IFITM2 promotes the expression of the type I IFN signaling pathway junction protein MDA5, which exerts antiviral effects [30]. QPCT, a glutamine amido cyclase, plays a key role in regulating the synthesis of peptide hormones, which may influence inflammatory responses [31]. Several previous studies have shown that glutamyl peptide transferase (QPCT), may be a biomarker for severe pancreatitis and that QPCT is strongly associated with the development of pancreatic and thyroid cancer [32,33]. Taken together, the above findings suggest that S100A11, IFITM2, and QPCT may also play key roles in the pathophysiologic process of sepsis. Compared with the above studies, the present study further revealed the potential biomarker roles of S100A11, IFITM2, and QPCT in sepsis, and verified the association of these genes in patient prognosis by Meta-analysis and survival curve analysis. These findings provide a basis for future clinical applications and also point the way to explore the specific mechanisms of these genes in sepsis management.

Among the current clinical markers of sepsis (including lactate, C-reactive protein, etc.), lactate is the most commonly used biomarker to recognize sepsis, but it is still not specific and does not realistically reflect changes in the condition [34]. This often influences sepsis treatment strategies and causes delays in sepsis treatment, which can lead to serious consequences. Therefore, S100A11, QPCT, and IFITM2, proposed in this study as new biomarkers for sepsis, aim to complement the inadequacy of conventional biomarkers by providing more specific and stable indicators that can reflect the patient’s condition at an earlier stage and more accurately, to optimize the diagnosis and personalized treatment strategies, and to improve the effectiveness of clinical management.

In summary, this study provides preliminary evidence for S100A11, IFITM2, and QPCT as novel sepsis biomarkers. Although this study provides preliminary evidence for S100A11, QPCT, and IFITM2 as potential biomarkers of sepsis, several limitations remain. First, the relatively small sample size may limit the generalizability of the results. Second, the lack of a randomized design may lead to confounding bias, which requires caution when interpreting the results. In addition, although we screened several key genes, other biomarkers (e.g., inflammatory factors, metabolic enzymes, etc.) that were not included may also play an important role in the development of sepsis. Therefore, future studies should expand the sample size and adopt a more rigorous experimental design to validate the role of these biomarkers in sepsis and reduce bias.

## Supporting Information

S1 Checklist Human participants research checklist. (DOCX)
